# Quality of Life of Young Gastric Cancer Survivors: An Interpretation in the Context of Our Society

**DOI:** 10.3390/medicina60010009

**Published:** 2023-12-20

**Authors:** Seung Soo Lee

**Affiliations:** 1 Department of Surgery, School of Medicine, Kyungpook National University, Daegu 41566, Republic of Korea; peterleess@knu.ac.kr; 2 Department of Surgery, Kyungpook National University Hospital, Daegu 41566, Republic of Korea

**Keywords:** competitive behavior, diarrhea, gastrectomy, quality of life, stomach neoplasm

## Abstract

*Background and Objectives*: This retrospective case-control study aimed to investigate the quality of life (QoL) of young gastric cancer survivors and determine what should be pursued to obtain the best QoL for them after surgery. *Materials and Methods*: Patients with stage I gastric cancer who underwent distal subtotal gastrectomy were included. The European Organization for Research and Treatment of Cancer Quality of Life questionnaires were used to assess QoL. The QoL outcomes of younger (age 30–49 years, *n* = 76) and older (age 50–69 years, *n* = 232) groups were compared preoperatively, 3 months, and 1 year postoperatively. *Results*: There was no statistically significant difference in the preoperative QoL except for the physical functioning scale, which favored the younger group (*p* = 0.020). This difference remained significant throughout the postoperative periods (3 months, *p* = 0.002; 1 year, *p* = 0.004). Better QoL was found for the younger group according to the body image scale (*p* = 0.031). However, it was limited to the 3-month postoperative assessment. Persistent QoL disadvantages for the younger group were revealed by the diarrhea scale at the 3-month (*p* = 0.007) and 1-year (*p* = 0.005) postoperative assessments. *Conclusions*: While young gastric cancer survivors enjoyed better QoL in physical functioning and body image, worse QoL was related to diarrhea after surgery. Despite ever-rising concerns about QoL deterioration in elderly patients in our aged society, young gastric cancer survivors also need QoL support.

## 1. Introduction

The age range of patients who undergo surgery for gastric cancer has expanded significantly over the last decade. While the increase in the number of elderly gastric cancer patients has been attributed to population aging for the past decades [1,2], the increased accessibility of the general population to endoscopic examinations has resulted in the detection of cancer at younger ages [3,4].

Gastric cancer and its treatments are known to strongly influence patients’ quality of life (QoL) [5,6,7]. QoL deteriorations in physical, functional, and psychological aspects have been reported at various points in the clinical process. Surgical treatment involves the loss of a significant portion of the stomach, mandating changes in the dietary habits of patients. Such behavior-oriented QoL consequences are known to last for a very long time [8].

Because population aging has been a recurrent social agenda, considerable efforts have been made to study the QoL of elderly patients. Thus, our knowledge on this subject has increased considerably. Although there are slight differences between studies, elderly patients often exhibited poor QoL scores compared to the general population [2]. According to the latest study, the extent of postoperative QoL change was not different between elderly and general populations. However, the absolute scores disfavored elderly patients [1].

Unlike the QoL of elderly patients, that of younger patients has not yet been addressed in depth. It might be tempting to assume that younger patients have better QoL based on previous findings of comparisons between general and elderly patients. However, this inference could be fallacious and oversimplified, ignoring the multifactorial traits of QoL [9,10,11]. QoL is known to be influenced by various external factors such as social, cultural, and informational elements [12]. An unexpected QoL resulting from these influences is not new [13]. In cases of breast cancer, poorer QoL outcomes among younger survivors related to fatigue, depression, and various functional aspects such as sexual, cognitive, and emotional functions have been reported [14,15]. It also has been reported that younger breast cancer survivors suffer poorer QoL related to financial difficulties.

Another trait of QoL is its relativity [11]. QoL represents the gap between reality and expectation. Among patients with similar surgical outcomes, those with higher expectations may have worse QoL [16]. Being older is associated with physiologic disadvantages. However, it is also accompanied by decreased personal and social expectations. Although being younger is associated with physiologic advantages, it is also associated with increased personal and social expectations. Hence, we must not rule out the possibility of unique QoL outcomes influenced by our contemporary society and culture.

The aim of this study was to investigate the QoL of young gastric cancer survivors and determine what should be pursued to achieve the best QoL for these patients in a developed Asian country. To achieve this, the peak age of competitive human behavior in social contexts (i.e., the age of 50) was used to classify gastric cancer survivors into younger and older groups [17].

## 2. Materials and Methods

### 2.1. Study Groups and Design

Gastric cancer patients aged 30 to 69 years who underwent a curative distal subtotal gastrectomy between 1 January 2010 and 30 September 2020 were included. A total of 560 patients who were confirmed to have stage I disease in accordance with the 8th edition of the Union for International Cancer Control (UICC) classification were considered eligible. QoL data from preoperative and 3-month and 1-year postoperative periods were available for 358 patients. Patients with comorbidities or histories of previous gastric surgery that could influence their QoL were considered ineligible. A total of 49 patients were excluded due to other malignancies (*n* = 13), previous gastric surgery (*n* = 4), cerebrovascular problems (*n* = 10), cardiac diseases (*n* = 6), endocrine problems (*n* = 6), psychological problems (*n* = 4), renal problems (*n* = 3), neurologic disease (*n* = 2), and a hepatic problem (*n* = 1). One patient with familial adenomatous polyposis who underwent a combined surgery of total proctocolectomy with ileal pouch–anal anastomosis was also excluded. After these exclusions, 308 patients with available data were finally analyzed.

The extent of gastrectomy as a curative treatment for gastric cancer is decided by the cancer location [18]. Distal subtotal gastrectomy is recommended for the curative resection of gastric cancer involving the lower part of the stomach. The principal surgical procedure with curative intent involves not only the resection of at least two-thirds of the stomach, but also D2 lymph node dissection. All patients with stage I gastric cancer in the lower part of the stomach underwent distal subtotal gastrectomy and D2 lymph node dissection. Both laparoscopic and open surgeries were recommended for stage I gastric cancer, and the patients were provided with a choice between them preoperatively. The choice of reconstruction method was based on the surgeon’s personal preference, and stapling devices were used for reconstruction. Postoperative adjuvant chemotherapy was delivered to patients with stage II/III gastric cancer to reduce recurrence. Thus, none of our patients underwent adjuvant chemotherapy. The influence of cancer cell differentiation on treatment planning was confined to endoscopic treatment, and it did not influence our surgical patients.

Competitive human behavior occurs in social contexts, and is known to peak around the age of 50 and drop thereafter [17]. On that basis, the study patients were classified into a younger group (30–49 years) and an older group (50–69 years) based on their age at surgery. There were 76 patients in the younger group and 232 patients in the older group. All gastric cancer patients underwent scheduled studies, educational sessions, and interviews according to our institutional protocol, and the protocol was applied to all patients equally. The patients were provided with identical information about postoperative changes, including postgastrectomy syndrome and dietary adjustments. QoL monitoring was a part of our standard institutional surveillance protocol, which included studies such as gastroscopy, computed tomography, and blood tests. Any deviation from the protocol for personal reasons was permitted.

### 2.2. QoL Assessment

Korean versions of the European Organization for Research and Treatment of Cancer QoL Questionnaire (EORTC QLQ) core (-C30) and gastric cancer-specific (-STO22) modules were used to assess QoL preoperatively, and at 3-months and 1-year postoperatively [19,20]. The QoL tools consisted of 52 items. For most items, responders are provided with available responses of 1, 2, 3, and 4 (representing “not at all”, “a little”, “quite a bit”, and “very much”, respectively). The exceptions are two items for global health status/QoL, which have available responses of 1 to 7. The patients were asked to respond to each item manually without clinician interference. The questionnaire was usually completed in about 20 min. Help was offered by designated personnel upon the request of those struggling with literacy or vision. Responders were allowed to skip questions if they chose. Information on comorbidities that may have influenced QoL was documented. The accuracy of the data was constantly monitored by two medical personnel. Preoperative QoL was assessed upon admission for surgery. Postoperative QoL was assessed at 3-month and 1-year postoperative follow-ups at the outpatient department.

Patients’ responses to QoL items were later transformed into 24 scale scores of 0–100 in accordance with the official scoring manual provided by the EORTC. Better QoL was considered to be reflected by lower scores, except for six scales (a global health status/QoL and five functional scales) on the EORTC QLQ-C30. Missed item responses were not regarded as ineligibility criteria for the overall assessment, as indicated by the scoring manual.

### 2.3. Statistical Analysis

Baseline demographic data and QoL scores for younger (30–49 years) and older (50–69 years) groups were compared preoperatively. Clavien–Dindo classification was used to classify postoperative complications. Postoperative 3-month and 1-year QoL scores were compared between the two groups.

Demographic data were compared using the chi-square test for categorical variables and Student’s *t*-test for continuous variables. Preoperative and 3-month and 1-year postoperative QoL outcomes were compared using Student’s *t*-test. Continuous variables are presented as means and standard deviations. The number of patients with valid responses allowing item–scale transformations was noted separately for each scale. A *p*-value of less than 0.05 was considered statistically significant. All statistical analyses were performed using IBM SPSS Statistics for Windows, version 25.0 (IBM Corp., Armonk, NY, USA).

## 3. Results

### 3.1. Demographic Characteristics of the Patients

The demographic characteristics of the patients in the younger (30–49 years) and older (50–69 years) groups are shown in Table 1. The mean age was 43.0 ± 3.9 years in the younger group and 59.7 ± 5.7 years in the older group. There was no significant difference in cancer stage or surgical procedures between the two groups. The rate of undifferentiated adenocarcinoma was higher in the younger group (76.3%) than in the older group (50.9%; *p* < 0.001). The number of retrieved lymph nodes was significantly higher in the younger group (38.2 ± 15.0) than in the older group (33.1 ± 13.5) (*p* = 0.006).

The rate of postoperative complications was 2.6% in the younger group and 5.2% in the older group, showing no significant difference between the two. All grade II complications, according the Clavien–Dindo classification, were related to short-term antibiotic therapy due to intraabdominal fluid collection or pulmonary complications. All three grade III complications were IIIa complications managed under local anesthesia. Two patients (one from each group) underwent radiologic intervention for abscess drainage. One patient from the older group underwent wound closure following a surgical site infection.

### 3.2. Patient Characteristics: Baseline QoL

There was no statistically significant difference in baseline QoL scores between the younger (70.4 ± 21.7) and older (64.7 ± 23.1) groups according to the global health status/QoL scale of the EORTC QLQ-C30 (Table 2). There were no significant QoL differences between the younger and older groups, except for QoL outcomes assessed by the physical functioning scale. According to the physical functioning scale, the QoL of the young group (90.8 ± 11.0) was significantly better than that of the older group (87.2 ± 13.3; *p* = 0.020).

### 3.3. Postoperative QoL Outcomes

Among the 24 scales of the EORTC QLQ -C30 and -STO22, only three scales revealed significant QoL differences (physical functioning, diarrhea, and body image scales) during the postoperative periods (Table 3). The preoperative QoL advantage in the younger group related to physical functioning persisted through the 3-month (*p* = 0.002) and 1-year postoperative periods (*p* = 0.004). The younger group exhibited QoL advantages in the body image scale (*p* = 0.031) at the 3-month postoperative assessment. However, this advantage was not seen at the 1-year postoperative assessment.

The diarrhea scale revealed QoL inferiority in the younger group at the 3-month postoperative assessment (*p* = 0.007). This inferiority persisted at the 1-year postoperative assessment (*p* = 0.005). Upon item analysis constituting diarrhea scale, those with “grave” item responses of 3 and 4 (“quite a bit”, and “very much”, respectively) were identified. At the 3-month postoperative period, there were 5 and 11 “grave” responses from younger and older groups, and 1 and 5 of them needed another dietary counseling due to lack of personal awareness of specific eating habits that had been causing diarrhea (20.0% and 45.5%), respectively. At the 1-year postoperative period, there were 8 and 13 “grave” responses from younger and older groups, and none and 9 of them needed another dietary counseling (0.0% and 46.2%), respectively.

## 4. Discussion

QoL advantages in young gastric cancer patients were not prominent, except in the physical functioning and body image scales. Considering that this QoL advantage in the physical functioning scale existed before surgery, better QoL in body image at the 3-month postoperative assessment was the sole and unprecedented advantage of young patients in terms of QoL. However, young patients experienced constant setbacks from worse QoL related to diarrhea.

Surgery for gastric cancer involves a significant loss of the food reservoir [21,22,23]. While an eventual return to the preoperative lifestyle is expected [24], modifications of dietary habits and their close monitoring are mandatory after surgery [25]. Dietary modifications include small frequent meals, slower eating, and separate consumption of liquid and solid food [26,27]. Unadjusted dietary habits after surgery likely result in frequent gastrointestinal symptoms such as nausea, vomiting, abdominal pain, and diarrhea. Dumping syndrome frequently occurs following surgery for gastric cancer, and is associated with rapid gastric emptying [28]. Its prevalence after partial gastrectomy is known to reach up to 50% [29], and Billroth II reconstruction is known to be associated with its higher incidence compared with other types of reconstruction. Most patients can be treated with dietary modification, and it may persist for years after surgery [30]. Early dumping syndrome is caused by the rapid transit of hyperosmolar chyme from the stomach into the duodenum, leading to shifting of fluid from the vasculature to the intestinal lumen [31]. This usually occurs within 10 to 30 min after a meal, and causes symptoms such as explosive diarrhea, abdominal pain, and tachycardia. Postvagotomy diarrhea is another type of diarrhea that affects gastric cancer survivors [28]. However, it is unrelated to oral intake, and resolves over the course of several months. Diarrhea associated with early dumping syndrome is often considered to be a sign of failing diet control after gastrectomy, and is being closely monitored.

In our hospital, all patients are provided multiple (scheduled and impromptu) dietary counseling sessions during the postoperative admission period and during follow-up visits to our outpatient department. There are four scheduled outpatient surveillance sessions (3-, 6-, 9-, and 12-month follow-ups) in the first year, and the interval between surveillances lengthens after the first year. The surveillance continues for five years after surgery. No exceptions were made regarding dietary counseling and monitoring. As there was no way for the patients to reach the same level of understanding at a given time, all patients were given handouts with printed guidelines for dietary modifications. Despite our efforts for successful communication regarding the need for dietary modifications, younger patients experienced more QoL setbacks related to diarrhea.

During our postoperative surveillance sessions, the cause of diarrhea is being investigated in all patients complaining of diarrhea. Our study design excluded patients with comorbidities that could have influences on their QoL during the pre- and postoperative periods. Therefore, those with diarrhea, which had been clinically judged as not being related to gastrectomy (e.g., food poisoning, inflammatory diarrhea), should have been excluded from this study. After identifying patients specifically suffering from diarrhea associated with early dumping syndrome, the level of awareness for dietary modification was evaluated. Surprisingly, the percentage of patients who suffered diarrhea due to lack of personal awareness of specific eating habits causing diarrhea was much smaller in younger patients.

The QoL of young gastric cancer survivors has not been studied in depth. Very few reports on the QoL of gastric cancer patients using the word “young” to name a group have been published. However, the word has been used merely to indicate a control group for elderly gastric cancer patients [32]. Broadening our search to all types of cancer revealed a recent report on the QoL of young cancer patients assessed by the EORTC QLQ-C30 [33]. Unlike our findings, the study suggested worse QoL on the diarrhea scale among older cancer patients. Patients with all types of cancer, with breast cancer comprising the larger proportion, were included. The inclusion of patients whose illness or treatment had less to do with postoperative dietary modification may have caused such a difference in results.

However, a previous study hinted at diarrhea-related QoL worsening among young gastric cancer survivors [2]. The study investigated the QoL of elderly gastric cancer patients. The mean age of the control group was set to the mid-50s, which was somewhat younger than usual, and diarrhea-related QoL inferiority of the control group against an elderly group was displayed. The age of the younger group in our study was further adjusted to those in their 30s and 40s, and their diarrhea-related QoL inferiority remained very prominent throughout the study period. However, some differences in outcomes were seen as well. These outcomes in several scales, such as physical functioning, role functioning, dyspnea, and dry mouth a year after surgery, suggest persistent QoL advantages in younger patients. In contrast, physical functioning was the only scale in our study with a persistent QoL advantage in younger patients. This may have been due to the differences in older patient age groups (≥70 years vs. 50–69 years), with our older group being much younger.

One might attempt to provide reasoning based on the notion that constipation increases with age [34,35,36], and that diarrhea decreases with age [37]. Although our cross-sectional comparisons at each postoperative period might seem to support this logic, the idea is not supported in its entirety due to the lack of baseline preoperative QoL differences in constipation and diarrhea. A more logical explanation becomes available once we shift our focus from the biological aspect of aging to the social aspect of QoL. Although QoL itself is known to be very personal, it is still within the context of culture and value systems [11,12]. Younger patients who are likely to be more socially active might have more quick meals to allow for a prompt return to social activities. They might also encounter ongoing problems with social meals as they try to keep up with their peers. As we live in a culture where higher sociality is expected from younger individuals, it is highly reasonable to assume that younger patients might be trading sociality for postoperative dietary modifications. This might have led to worse QoL related to diarrhea in younger patients. Our institutional protocol for gastric cancer patients was equally applied to all patients. All educational session and interview content was the same. Despite the same clinical input, younger patients reported different subjective outcomes. Therefore, future research needs to concentrate on the processing of input by younger patients.

By turning our attention to the social aspect of QoL, it becomes very reasonable to regard young gastric cancer survivors as being in constant need of reminders to keep up with modified diets and prescriptions for antidiarrheal medicine. It also triggers a new concept of QoL management for young gastric cancer survivors. They need to have small and frequent meals, as do other survivors, while their social lives need to remain unaffected. Previous efforts have been made to fill nutritional needs with nutrient-packed mini-meals, such as manufactured oral nutritional formulas or supplements [38,39,40]. However, the targets were mostly elderly patients. Based on our findings, redirecting the recipients of oral nutritional supplements to socially active young gastric cancer survivors might be needed. In our institution, manufactured oral nutritional formula has been prescribed to outpatients mostly upon their request. While most requests seem to come from facing temporary dietary difficulties, there are occasions when we suspect that products are being regularly consumed as convenient between-meal foods to minimize social interference from surgically-necessitated frequent meals.

An explanation is needed for the significant difference in the number of retrieved lymph nodes. All patients were treated by the same protocol, and all underwent D2-extent lymphadenectomy regardless of open or laparoscopic approach. Despite the lack of differences in surgical intent, younger patients had a significantly higher number of retrieved lymph nodes. Considering a higher percentage of undifferentiated adenocarcinoma among younger patients, a higher number of retrieved lymph nodes among them may have reflected an additional effort by pathologists, harvesting more lymph nodes from surgical pathology specimens from patients with preoperatively confirmed undifferentiated adenocarcinoma. It has been reported that specimen handling by pathologists is strongly associated with the number of lymph node harvested [41].

The strength of this study was that it provided a reason to direct attention to the QoL of young gastric cancer survivors, since our latest concerns have been skewed toward the QoL of elderly patients. The revelation of difficulty and frustration related to diarrhea among young gastric cancer survivors is a key finding of this study. Upon repeated complaints of diarrhea from a socially active young gastric cancer survivor, we need to verify if the patient has been trading postoperative diet control for social competitiveness. If it is suspected that the patient is determined to maintain the same diet pattern as peers, even if that may eventually lead to diarrhea, there is no need to reserve antidiarrheal medications as a last resort. Efforts to provide constant reminders of dietary modifications in the form of shorter-interval follow-ups should be made. In addition to medical efforts of controlling already occurred symptom (i.e., diarrhea), its preventive measures involving maximizing the preservation of social competitiveness while minimizing the challenges of diet modification should be devised. Prescription of manufactured oral nutritional supplements to young gastric cancer survivors with repeated complaints of diarrhea is highly recommended.

This study was not without limitations. First, we built our hypothesis based on the social tendency of the young population to have more social activities. Without actual information about their social status, more conclusive evidence must be provided. Subgroup analyses based on actual social activity status should be able to provide clearer evidence. Thus, larger study groups need to be built with multi-institutional effort. Secondly, we did not have information on the amount and frequency of diarrhea, quantified by a uniform measure. Early dumping-associated diarrhea is an episodic event occurring shortly after deviation from postgastrectomy diet protocol. Despite the effort for excluding patients with diarrhea unrelated to early dumping syndrome, having objective indicators available for background information would allow better justification of the discussion. Our findings represent a significant basis for future prospective studies focusing on diarrhea among younger gastric cancer survivors. Third, comparison groups were asymmetrical in terms of sample size. The sample size of the older group was about three times that of the younger group. The younger age was set as patients in their 30s and 40s by referring to a previous QoL study on elderly patients with incidental findings of QoL setback in a younger control group with a mean age in the 50s [2]. To decide if this finding was a singular QoL issue for young patients, we pushed the age limit to 30s and 40s, and compared their QoL against the QoL of the controls (older group). Since our society has already turned into an aged society, asymmetry in sample size by age group is inevitable. Designing matched comparisons based on a larger pool of data with multiple variables should increase statistical significance. Finally, there was a risk of the concealment of more severe QoL deterioration in young gastric cancer patients with diarrhea. This study was conducted in a country that had already become an aged society, and requests for maintaining social competitiveness even at an advanced age may be rampant. Given that, our older control group may have included some socially competitive individuals who traded diet modifications for social competitiveness.

## 5. Conclusions

While young gastric cancer survivors enjoyed better QoL in physical functioning and body image, worse QoL in patients with diarrhea persisted a year after surgery. Young gastric cancer survivors could be trading social competitiveness for dietary modifications after gastrectomy, and countermeasures such as strict dietary monitoring and the prescription of oral nutritional supplements must be applied. The exact relationship between higher will/demand for social activities and postoperative dietary adherence in young patients needs to be explored in the future.

## Figures and Tables

**Table 1:** Characteristics of patients in the younger (30–49 years) and older (50–69 years) groups.

Characteristics	Age Group (Years)		*p*-Value
	30–49 (*n* = 76)	50–69 (*n* = 232)	
Sex			0.206
Female	33 (43.4)	82 (35.3)	
Male	43 (56.6)	150 (64.7)	
Age (year)	43.0 ± 3.9	59.7 ± 5.7	<0.001 *
Body mass index (kg/m^2^)	23.5 ± 3.2	23.9 ± 2.8	0.418
Stage ^†^			0.616
IA	68 (89.5)	212 (90.9)	
IB	8 (10.5)	20 (9.1)	
Pathology			<0.001 *
Differentiated	18 (23.7)	114 (49.1)	
Undifferentiated	58 (76.3)	118 (50.9)	
Route of surgery			0.686
Open	53 (69.7)	156 (67.2)	
Laparoscopic	23 (30.3)	76 (32.8)	
Anastomosis			0.380
Billroth I	71 (93.4)	209 (90.1)	
Billroth II	5 (6.6)	23 (9.9)	
Number of retrieved lymph nodes	38.2 ± 15.0	33.1 ± 13.5	0.006 *
Postoperative hospital stay, days	7.4 ± 3.5	7.7 ± 3.7	0.545
Postoperative complications			0.356
No	74 (97.4)	220 (94.8)	
Yes ^‡^	2 (2.6)	12 (5.2)	
Grade I	1	6	
Grade II	0	4	
Grade III	1	2	
Grade IV	0	0	
Grade V	0	0	

Values are presented as number only, number (%), or mean ± standard deviation. * *p* < 0.05, significant difference between the two age groups. ^†^ Stage grouping was based on the 8th edition of the Union for International Cancer Control classification. ^‡^ Complications were classified according to the Clavien–Dindo classification.

**Table 2:** Baseline quality of life of younger (30–49 years) and older (50–69 years) groups.

Variable	30–49 Years		50–69 Years		*p*-Value
	R	Mean ± SD	R	Mean ± SD	
EORTC QLQ-C30					
Global health status/QoL ^†^	71	70.4 ± 21.7	219	64.7 ± 23.1	0.068
Functional scale ^†^					
Physical functioning	76	90.8 ± 11.0	229	87.2 ± 13.3	0.020 *
Role functioning	76	91.9 ± 15.5	229	92.4 ± 13.8	0.773
Emotional functioning	76	79.8 ± 18.7	229	81.6 ± 18.4	0.478
Cognitive functioning	76	90.6 ± 15.2	229	88.4 ± 14.1	0.261
Social functioning	76	86.6 ± 20.0	229	86.5 ± 19.4	0.951
Symptom scales/items ^‡^					
Fatigue	76	22.6 ± 17.5	229	19.0 ± 17.6	0.120
Nausea and vomiting	76	7.9 ± 15.3	229	6.3 ± 12.7	0.356
Pain	76	8.6 ± 16.0	229	7.9 ± 13.4	0.740
Dyspnea	76	6.6 ± 17.2	228	10.5 ± 19.4	0.096
Insomnia	76	14.9 ± 22.0	227	13.1 ± 21.3	0.518
Appetite loss	76	14.0 ± 21.3	229	10.0 ± 19.8	0.136
Constipation	75	15.1 ± 22.8	227	13.1 ± 20.8	0.473
Diarrhea	75	15.6 ± 20.7	228	11.5 ± 18.7	0.119
Financial difficulties	75	16.9 ± 25.3	220	14.1 ± 22.9	0.375
EORTC QLQ-STO22 ^‡^					
Dysphagia	76	5.6 ± 11.4	228	5.5 ± 9.9	0.943
Pain	76	15.2 ± 15.4	228	13.5 ± 14.6	0.373
Reflux	76	10.6 ± 13.5	228	10.9 ± 15.0	0.890
Eating restrictions	76	6.7 ± 11.4	228	6.8 ± 10.1	0.916
Anxiety	76	23.4 ± 19.1	228	23.3 ± 19.6	0.977
Dry mouth	76	18.9 ± 27.4	228	20.0 ± 25.5	0.734
Taste	76	7.5 ± 16.9	225	6.5 ± 16.0	0.663
Body image	76	13.2 ± 20.4	224	15.6 ± 23.2	0.410
Hair loss	27	19.8 ± 31.0	73	5.5 ± 9.9	0.398

QoL = quality of life, R = number of responders, SD = standard deviation, EORTC QLQ = European Organization for Research and Treatment of Cancer quality of life questionnaire. * *p* < 0.05, significant difference between the two age groups. ^†^ Higher scores represent better QoL. ^‡^ Higher scores represent worse QoL.

**Table 3:** Postoperative quality of life outcomes of younger (30–49 years) and older (50–69 years) groups assessed 3 months and 1 year after surgery.

Variable	Postoperative Period									
	3-Months					1-Year				
	30–49 Years		50–69 Years		*p*-Value	30–49 Years		50–69 Years		*p*-Value
	R	Mean ± SD	R	Mean ± SD		R	Mean ± SD	R	Mean ± SD	
EORTC QLQ-C30										
Global health status/QoL ^†^	68	72.7 ± 22.3	191	67.4 ± 22.9	0.099	69	76.2 ± 19.8	193	72.7 ± 24.2	0.232
Functional scale ^†^										
Physical functioning	70	87.5 ± 10.2	219	82.5 ± 15.3	0.002 *	75	90.0 ± 10.8	232	85.6 ± 12.6	0.004 *
Role functioning	70	87.6 ± 16.7	220	82.7 ± 20.7	0.073	75	89.8 ± 16.0	232	86.0 ± 17.7	0.101
Emotional functioning	70	84.8 ± 16.4	220	85.6 ± 18.5	0.741	75	82.4 ± 19.7	232	86.0 ± 15.5	0.107
Cognitive functioning	70	87.9 ± 14.4	220	87.4 ± 14.6	0.829	75	86.4 ± 17.3	231	85.7 ± 16.2	0.738
Social functioning	70	87.1 ± 18.4	218	85.8 ± 19.4	0.605	75	87.8 ± 22.3	229	89.2 ± 16.4	0.567
Symptom scales/items ^‡^										
Fatigue	70	27.5 ± 18.5	219	27.2 ± 19.8	0.936	75	23.6 ± 19.6	232	23.7 ± 18.8	0.952
Nausea and vomiting	70	6.4 ± 10.7	220	8.5 ± 15.3	0.297	75	8.7 ± 13.2	232	8.0 ± 13.3	0.726
Pain	70	10.2 ± 12.1	219	11.6 ± 15.6	0.490	75	8.0 ± 13.5	232	11.4 ± 16.2	0.078
Dyspnea	69	8.7 ± 15.8	218	10.2 ± 19.8	0.554	75	8.4 ± 19.8	231	11.1 ± 18.6	0.289
Insomnia	70	13.3 ± 18.3	219	12.0 ± 23.1	0.665	75	11.6 ± 20.9	231	12.3 ± 21.5	0.803
Appetite loss	70	12.9 ± 20.7	218	13.1 ± 21.2	0.920	75	7.6 ± 17.0	229	10.8 ± 17.4	0.159
Constipation	70	17.1 ± 23.2	219	15.4 ± 23.5	0.583	75	16.4 ± 21.5	232	15.4 ± 20.5	0.698
Diarrhea	68	25.5 ± 20.9	219	17.7 ± 20.8	0.007 *	75	26.7 ± 21.9	231	18.5 ± 21.4	0.005 *
Financial difficulties	70	17.1 ± 27.1	219	14.0 ± 22.3	0.332	75	12.0 ± 21.7	230	13.0 ± 20.3	0.704
EORTC QLQ-STO22 ^‡^										
Dysphagia	70	10.6 ± 10.7	218	12.1 ± 12.6	0.387	76	8.2 ± 11.9	228	8.3 ± 10.7	0.960
Pain	70	17.9 ± 14.1	218	17.3 ± 16.1	0.806	76	15.3 ± 13.4	229	14.8 ± 14.3	0.797
Reflux	70	8.9 ± 12.2	218	10.0 ± 15.1	0.588	76	8.5 ± 12.8	229	11.4 ± 14.7	0.129
Eating restrictions	70	15.6 ± 15.6	218	14.0 ± 15.6	0.465	76	14.1 ± 15.0	228	11.6 ± 12.8	0.161
Anxiety	70	28.1 ± 20.4	218	27.7 ± 20.8	0.876	76	27.2 ± 21.2	229	26.7 ± 20.1	0.844
Dry mouth	70	17.6 ± 23.9	217	16.3 ± 21.8	0.663	76	15.4 ± 20.7	227	19.7 ± 22.9	0.146
Taste	70	7.6 ± 16.2	218	8.7 ± 16.9	0.635	75	4.4 ± 11.4	227	6.8 ± 14.1	0.155
Body image	69	18.8 ± 22.5	218	26.5 ± 26.4	0.031 *	76	18.4 ± 25.2	227	23.5 ± 27.6	0.158
Hair loss	20	28.3 ± 32.9	74	17.1 ± 22.9	0.082	34	30.4 ± 30.0	99	22.6 ± 25.1	0.138

QoL = quality of life, R = number of responders, SD = standard deviation, EORTC QLQ = European Organization for Research and Treatment of Cancer quality of life questionnaire. * *p* < 0.05, significant difference between the two age groups. ^†^ Higher scores represent better QoL. ^‡^ Higher scores represent worse QoL.

## Data Availability

Data are available upon reasonable request.

## References

[1] Jung J.H., Lee S.S., Chung H.Y. (2022). Quality of life changes in elderly patients after gastrectomy: Perspective of an aged Asian society in the 2010s. Ann. Surg. Treat. Res..

[2] Han D.S., Ahn J., Ahn H.S. (2021). Are the elderly patient’s changes in the health-related quality of life one year after gastrectomy for stomach cancer different from those in young patients?. Ann. Surg. Treat. Res..

[3] Katai H., Sasako M., Sano T., Maruyama K. (1996). Gastric carcinoma in young adults. Jpn. J. Clin. Oncol..

[4] Hallissey M.T., Allum W.H., Jewkes A.J., Ellis D.J., Fielding J.W. (1990). Early detection of gastric cancer. BMJ.

[5] Yu W., Park K.B., Chung H.Y., Kwon O.K., Lee S.S. (2016). Chronological changes of quality of life in long-term survivors after gastrectomy for gastric cancer. Cancer Res. Treat..

[6] Kim A.R., Cho J., Hsu Y.J., Choi M.G., Noh J.H., Sohn T.S., Bae J.M., Yun Y.H., Kim S. (2012). Changes of quality of life in gastric cancer patients after curative resection: A longitudinal cohort study in Korea. Ann. Surg..

[7] Lee S.S., Chung H.Y., Yu W. (2010). Quality of life of long-term survivors after a distal subtotal gastrectomy. Cancer Res. Treat..

[8] Lee S.S., Chung H.Y., Kwon O.K., Yu W. (2016). Long-term quality of life after distal subtotal and total gastrectomy: Symptom- and behavior-oriented consequences. Ann. Surg..

[9] Bottomley A. (2002). The cancer patient and quality of life. Oncologist.

[10] WHOQOL Group (1993). Study protocol for the World Health Organization project to develop a quality of life assessment instrument (WHOQOL). Qual. Life Res..

[11] Calman K.C. (1984). Quality of life in cancer patients—An hypothesis. J. Med. Ethics.

[12] Felce D., Perry J. (1995). Quality of life: Its definition and measurement. Res. Dev. Disabil..

[13] Lee S.S., Chung H.Y., Kwon O.K. (2020). Information-stressors and cancer patients’ quality of life: Responses to deviant information-stressors due to pre-postoperative stage discordance. Chonnam Med. J..

[14] Champion V.L., Wagner L.I., Monahan P.O., Daggy J., Smith L., Cohee A., Ziner K.W., Haase J.E., Miller K.D., Pradhan K. (2014). Comparison of younger and older breast cancer survivors and age-matched controls on specific and overall quality of life domains. Cancer.

[15] Maurer T., Thöne K., Obi N., Jung A.Y., Behrens S., Becher H., Chang-Claude J. (2021). Health-related quality of life in a cohort of breast cancer survivors over more than 10 years post-diagnosis and in comparison to a control cohort. Cancers.

[16] Lee S.S., Ryu S.W., Kim I.H., Sohn S.S. (2012). Quality of life beyond the early postoperative period after laparoscopy-assisted distal gastrectomy: The level of patient expectation as the essence of quality of life. Gastric Cancer.

[17] Mayr U., Wozniak D., Davidson C., Kuhns D., Harbaugh W.T. (2012). Competitiveness across the life span: The feisty fifties. Psychol. Aging.

[18] Japanese Gastric Cancer Association (2023). Japanese gastric cancer treatment guidelines 2021 (6th edition). Gastric Cancer.

[19] Aaronson N.K., Ahmedzai S., Bergman B., Bullinger M., Cull A., Duez N.J., Filiberti A., Flechtner H., Fleishman S.B., de Haes J.C. (1993). The European Organization for Research and Treatment of Cancer QLQ-C30: A quality-of-life instrument for use in international clinical trials in oncology. J. Natl. Cancer Inst..

[20] Yun Y.H., Park Y.S., Lee E.S., Bang S.M., Heo D.S., Park S.Y., You C.H., West K. (2004). Validation of the Korean version of the EORTC QLQ-C30. Qual. Life Res..

[21] de Calan L., Portier G., Ozoux J.P., Rivallain B., Perrier M., Brizon J. (1988). Carcinoma of the cardia and proximal third of the stomach. Results of surgical treatment in 91 consecutive patients. Am. J. Surg..

[22] Bozzetti F., Marubini E., Bonfanti G., Miceli R., Piano C., Gennari L. (1999). Subtotal versus total gastrectomy for gastric cancer: Five-year survival rates in a multicenter randomized Italian trial. Italian Gastrointestinal Tumor Study Group. Ann. Surg..

[23] Yoo C.H., Sohn B.H., Han W.K., Pae W.K. (2004). Long-term results of proximal and total gastrectomy for adenocarcinoma of the upper third of the stomach. Cancer Res. Treat..

[24] Calomino N., Malerba M., Tanzini G. (1998). Gastrectomia totale e qualità della vita [Total gastrectomy and quality of life]. Minerva Chir..

[25] Calomino N., Malerba M., Palasciano G., Cappelli A., Oliva G., Salvestrini F., Tanzini G. (1998). Gastrectomia totale e malnutrizione [Total gastrectomy and malnutrition]. Minerva Chir..

[26] Braga M., Zuliani W., Foppa L., Di Carlo V., Cristallo M. (1988). Food intake and nutritional status after total gastrectomy: Results of a nutritional follow-up. Br. J. Surg..

[27] Samrat R., Naimish M., Samiran N. (2020). Post-gastrectomy complications—An overview. Chirurgia.

[28] Davis J.L., Ripley R.T. (2017). Postgastrectomy syndromes and nutritional considerations following gastric surgery. Surg. Clin. North Am..

[29] Chaves Y.D., Destefani A.C. (2016). Pathophysiology, diagnosis and treatment of dumping syndrome and its relation to bariatric surgery. Arq. Bras. Cir. Dig..

[30] Chaimoff C., Dintsman M., Tiqva P. (1972). The long-term fate of patients with dumping syndrome. Arch. Surg..

[31] Bolton J.S., Conway W.C. (2011). Postgastrectomy syndromes. Surg. Clin. North Am..

[32] Mohri Y., Yasuda H., Ohi M., Tanaka K., Saigusa S., Okigami M., Shimura T., Kobayashi M., Kusunoki M. (2015). Short- and long-term outcomes of laparoscopic gastrectomy in elderly patients with gastric cancer. Surg. Endosc..

[33] Lehmann J., Riedl D., Nickels A., Sanio G., Hassler M., Rumpold G., Holzner B., Licht T. (2023). Associations of age and sex with the efficacy of inpatient cancer rehabilitation: Results from a longitudinal observational study using electronic patient-reported outcomes. Cancers.

[34] Gallegos-Orozco J.F., Foxx-Orenstein A.E., Sterler S.M., Stoa J.M. (2012). Chronic constipation in the elderly. Am. J. Gastroenterol..

[35] Gallagher P., O’Mahony D. (2009). Constipation in old age. Best Pract. Res. Clin. Gastroenterol..

[36] Johanson J.F., Sonnenberg A., Koch T.R. (1989). Clinical epidemiology of chronic constipation. J. Clin. Gastroenterol..

[37] Nolte S., Liegl G., Petersen M.A., Aaronson N.K., Costantini A., Fayers P.M., Groenvold M., Holzner B., Johnson C.D., Kemmler G. (2019). General population normative data for the EORTC QLQ-C30 health-related quality of life questionnaire based on 15,386 persons across 13 European countries, Canada and the Unites States. Eur. J. Cancer.

[38] Li M., Zhao S., Wu S., Yang X., Feng H. (2021). Effectiveness of oral nutritional supplements on older people with anorexia: A systematic review and meta-analysis of randomized controlled trials. Nutrients.

[39] Ashkenazi I., Rotman D., Amzalleg N., Graif N., Khoury A., Ben-Tov T., Steinberg E. (2022). Efficacy of oral nutritional supplements in patients undergoing surgical intervention for hip fracture. Geriatr. Orthop. Surg. Rehabil..

[40] Lai W.Y., Chiu Y.C., Lu K.C., Huang I.T., Tsai P.S., Huang C.J. (2021). Beneficial effects of preoperative oral nutrition supplements on postoperative outcomes in geriatric hip fracture patients: A PRISMA-compliant systematic review and meta-analysis of randomized controlled studies. Medicine.

[41] Storli K., Lindboe C.F., Kristoffersen C., Kleiven K., Søndenaa K. (2011). Lymph node harvest in colon cancer specimens depends on tumour factors, patients and doctors, but foremost on specimen handling. APMIS.

